# Estrogen Regulates Glucose Metabolism in Cattle Neutrophils Through Autophagy

**DOI:** 10.3389/fvets.2021.773514

**Published:** 2021-11-29

**Authors:** Xinbo Wang, Yuming Zhang, Yansong Li, Mingyu Tang, Qinghua Deng, Jingdong Mao, Liyin Du

**Affiliations:** Clinical Veterinary Laboratory, College of Animal Science and Technology, Inner Mongolia MINZU University, Tongliao, China

**Keywords:** estrogen, glucose metabolism, autophagy, ATP, polymorphonuclear neutrophils, cattle

## Abstract

Hypoglycemia resulting from a negative energy balance (NEB) in periparturient cattle is the major reason for a reduced glycogen content in polymorphonuclear neutrophils (PMNs). The lack of glycogen induces PMNs dysfunction and is responsible for the high incidence of perinatal diseases. The perinatal period is accompanied by dramatic changes in sex hormones levels of which estrogen (17β-estradiol, E2) has been shown to be closely associated with PMNs function. However, the precise regulatory mechanism of E2 on glucose metabolism in cattle PMNs has not been elucidated. Cattle PMNs were cultured in RPMI 1640 with 2.5 (LG), 5.5 (NG) and 25 (HG) mM glucose and E2 at 20 (EL), 200 (EM) and 450 (EH) pg/mL. We found that E2 maintained PMNs viability in different glucose conditions, and promoted glycogen synthesis by inhibiting PFK1, G6PDH and GSK-3β activity in LG while enhancing PFK1 and G6PDH activity and inhibiting GSK-3β activity in HG. E2 increased the ATP content in LG but decreased it in HG. This indicated that the E2-induced increase/decrease of ATP content may be independent of glycolysis and the pentose phosphate pathway (PPP). Further analysis showed that E2 promoted the activity of hexokinase (HK) and GLUT1, GLUT4 and SGLT1 expression in LG, while inhibiting GLUT1, GLUT4 and SGLT1 expression in HG. Finally, we found that E2 increased LC3, ATG5 and Beclin1 expression, inhibited p62 expression, promoting AMPK-dependent autophagy in LG, but with the opposite effect in HG. Moreover, E2 increased the Bcl-2/Bax ratio and decreased the apoptosis rate of PMNs in LG but had the opposite effect in HG. These results showed that E2 could promote AMPK-dependent autophagy and inhibit apoptosis in response to glucose-deficient environments. This study elucidated the detailed mechanism by which E2 promotes glycogen storage through enhancing glucose uptake and retarding glycolysis and the PPP in LG. Autophagy is essential for providing ATP to maintain the survival and immune potential of PMNs. These results provided significant evidence for further understanding the effects of E2 on PMNs immune potential during the hypoglycemia accompanying perinatal NEB in cattle.

## Introduction

Negative energy balance (NEB) during the perinatal period in cattle increases the incidence of mammary gland and uterine infectious diseases, such as mastitis, uteritis, retained fetal membranes (RFM), and endometritis. These diseases are associated with polymorphonuclear neutrophils (PMNs) dysfunction induced by the dramatic changes in steroid hormone levels. Studies have found that changes in estrogen (17β-estradiol, E2), a steroid hormone, may be responsible for the reduced immune response of PMNs before and after parturition (1). E2 in periparturient cows is known to increase in the 2 weeks immediately before parturition, rising from a basal level of 20 pg/mL to a peak of 450 pg/mL, then rapidly declining to the basal level (2). Many studies have confirmed that abnormal E2 levels may lead to perinatal diseases. For example, the level of E2 in cows that suffered from RFM was higher than that of normal cows 6 days before parturition (3, 4). Cows suffering from subclinical mastitis showed low circulating E2 levels (5). These results suggest that normal levels of E2 could relieve immunosuppression in periparturient cattle.

Previous studies have shown that ATP in PMNs is mainly produced by glucose metabolism, and elevated ATP levels are conducive to normal PMNs function during parturition (6). Regarding glucose metabolism in PMNs, glucose is first phosphorylated by hexokinase (HK) to produce glucose 6-phosphate (G6P), after which G6P is used for ATP and NADPH production via glycolysis and the pentose phosphate pathway (PPP) and glycogen synthesis (7). Hypoglycemia after parturition affects the maintenance of optimal intracellular glycogen levels and PMNs function, especially in cows suffering from uterine or mammary disease as described above. E2 has been shown to promote glycogen synthesis in various tissues and cells by regulating glycogen synthase kinase-3β (GSK-3β), for example, in the uterus (8), astrocytes (9), and neurons (10). However, to the best of our knowledge, there is no research on E2 regulation of glycogen synthesis in cattle PMNs. Reports have shown that glycolysis is the major pathway for ATP generation in PMNs, while the PPP is involved in NADPH generation (11). Both these pathways, together with the glycogen synthesis pathway, play important roles in glucose metabolism (12). As one of the most important regulatory enzymes in glycolysis, phosphofructosekinase-1 (PFK1) catalyzes the conversion of fructose-6-phosphate to fructose-1,6-diphosphate in response to cellular energy requirements, while glucose-6-phosphate dehydrogenase (G6PDH), the key enzyme in the PPP, fuels NADPH to produce superoxide. Numerous studies have shown that E2 plays an important regulatory role in glycolysis, the PPP, and other pathways of glucose metabolism in various types of cells, including MCF-7 breast cancer (13), uterine (14), and HeLa cervical cancer cells (15). Unfortunately, the specific mechanism of E2 action on glucose metabolism in PMNs is still unclear. It is, thus, worthwhile to improve our understanding of the role of glucose deficiency and E2 level on the immune potential of PMNs.

It is well-known that low extracellular glucose levels result in a deficiency in glucose uptake and utilization, and the glucose transport system of PMNs is responsible for the uptake of circulating glucose (16). To date, two glucose transporter families have been identified, including the main GLUT superfamily (GLUTs) and the sodium-glucose co-transporter SGLT family (SGLTs) (17). As the members of the GLUTs, the expression of GLUT1 and GLUT4 varies with PMNs biological conditions with glucose mainly transported across cell membranes by GLUT1 under physiological conditions (18). Meanwhile, SGLTs transport glucose through a secondary active transport mechanism, which promotes glucose uptake by using the sodium concentration gradient established by the Na^+^/K^+^-ATPase pump (19). As a member of SGLTs, the SGLT1 is mainly expressed in the kidney, heart and trachea. SGLT1 expression and its relationship to GLUT1/4 in cattle PMNs under changed glucose environments is completely unknown.

AMP-activated protein kinase (AMPK) senses available energy in cells by binding directly to ATP. Activated AMPK increases the translocation of glucose transporters and promotes ATP preservation and production. Once ATP production pathways such as glycolysis, fatty acid oxidation (FAO) and oxidative phosphorylation (OXPHOS) are activated, pathways involving ATP consumption, such as protein synthesis, fatty acid synthesis, gluconeogenesis and glycogen synthesis pathways, are reduced. However, reports on the role of AMPK in glucose metabolism are contradictory. Some studies have shown that activated AMPK phosphorylates key proteins of multiple pathways such as glycolysis, leading to enhanced catabolism and reduced anabolism (20, 21). Other studies have shown that AMPK activation is associated with glycogen accumulation rather than glycogen consumption (22, 23). Therefore, this study aimed to clarify the role of AMPK in the glucose metabolism of cattle PMNs.

The Bcl-2 superfamily both promotes and inhibits apoptosis, and the balance pro-apoptotic and anti-apoptotic proteins, such as Bax and Bcl-2, respectively, is critical for determining the survival time of mature PMNs. Previous study have found that the spontaneous apoptosis of human PMNs in the absence of sufficient nutrients could be inhibited by increased glucose *in vitro* (24). As is well-known, autophagy can supplement anabolic substrates and energy under low energy conditions by degrading internal cellular components (25) and autophagy markers including ATG5, Beclin1, LC3 and p62 play key roles in autophagy initiation in various cells (26). Studies have shown that E2 can promote autophagy, delay senescence (27) and inhibit apoptosis (28). Although increasing evidence shows that E2 can regulate cellular glucose metabolism, there are few studies on the mechanism of E2 regulation of PMNs autophagy.

The effects of E2 on the immune potential of cattle PMNs and its association with glucose levels have not been studied. Here, we investigated the effect and mechanism of E2 on glucose metabolism through regulation glucose uptake and utilization, to determine whether E2 enhances PMNs immune potential by activating autophagy under low-glucose conditions. This study provided valuable new perspectives on how E2 controls the immune potential of PMNs in cattle suffering from perinatal NEB.

## Materials and Methods

### Animals

All experiments were conducted in accordance with relevant guidelines and regulations. The current study was conducted at the Inner Mongolia University for Nationalities in Tongliao, China. Jugular venipuncture blood samples were collected from the ovariectomized Chinese Simmental cattle aged about 2 years.

### Isolation and Culture of PMNs

The blood was collected by jugular vein puncture in cattle into 50 mL centrifuge tubes containing 0.1 mL heparin (Gentihold) as an anticoagulant. Heparinized blood was diluted with equal amounts of 1 × PBS, placed on the Percoll (GE Healthcare) separation solution, and centrifuged at 800 × g for 15 min. After removal of the plasma, red blood cell and PMNs were collected, washed once with PBS, then the red blood cell lysates were added and centrifuged at 800 × g for 8 min. PMNs were washed once again with 1 × PBS and once with RPMI 1,640 medium (Procell), and then were resuspended in RPMI 1,640 medium (2.0 × 10^6^ cells/mL). The PMNs were incubated in RPMI 1,640 containing 10% fetal cattle serum (Hyclone, Logan, UT, USA) at 37°C and 5% CO_2_ for 45 min. After 45 min, the PMNs were cultured in fresh medium.

PMNs were incubated with different concentrations of 17β-estradiol (Sigma) and glucose (Sigma) for specified times. The concentrations of E2 were 20 pg/mL (EL), 200 pg/mL (EM) and 450 pg/mL (EH). The glucose concentrations were 2.5 mM (LG), 5.5 mM (NG) and 25 mM (HG). PMNs were incubated with EH for 6 h under LG, NG and HG conditions. The PMNs were then collected for subsequent tests.

### Cell Counting Kit-8 Assay

The viability of PMNs cells was determined with a Cell Counting Kit-8 detection kit (CCK-8; Biosharp, China) in accordance with the manufacturer's protocol. Briefly, the cells were seeded into 96-well plates at the density of 5 × 10^3^ cells per well. At the indicated time point, 10 μL of CCK-8 solution was added, and PMNs were incubated at 37°C in a 5% CO_2_ incubator for 0, 2, 4, 6, 8, and 12 h at different concentrations of E2 and glucose. The absorbance was measured at 450 nm under an automatic microplate reader (Multiskan Spectrum; Thermo Scientific, USA).

### Biochemical Analyses

Biochemical analysis was used to detect activities of different enzymes in PMNs cultured with different concentrations of E2 and glucose for 6 h. All biochemical tests, HK and G6PDH activity, and ATP and glycogen content, were performed using commercial test kits (Solarbio, Beijing, China) at 37°C in an automatic microplate reader (Multiskan Spectrum; Thermo Scientific). Biochemical analyses were conducted in strict accordance with the instructions of the kits.

### ELISA Analysis

PMNs cultured with different concentrations of E2 and glucose for 6 h were collected and the activity of PFK1 and GSK-3β in cells was detected by ELISA kit (SolarBio). ELISA analysis was conducted in strict accordance with the instructions of the kit.

### Western Blotting Analysis

The PMNs were treated for 6 h with 2.5, 5.5 and 25 mM glucose and EH. Total protein was extracted from PMNs with lysis buffer (Solarbio). The protein concentration was quantified using a BCA protein assay kit (Applygen). Then, the protein samples were separated by sodium dodecyl sulfate-polyacrylamide gel electrophoresis (SDS-PAGE) and transferred onto polyvinylidene fluoride (PVDF) membranes (Immobilon). After blocking with 5% BSA for 2 h, the membranes were blotted with 1:700 diluted primary antibodies against GLUT1 (Abcam, MA, USA), GLUT4 (Abcam), SGLT1 (Cell Signaling Technology, MA, USA), Beclin1 (Abcam), ATG5 (Abcam), p62 (Abcam), LC3 (Cell Signaling Technology), β-actin (Absin), AMPK (Abcam), p-AMPK (PL Laboratories, USA), Bax (Abcam), or Bcl-2 (Abcam) at 4°C overnight. The membrane was washed with Tris-buffered saline containing 0.1% Tween-20 (TBST), and the secondary antibodies (Cell Signaling Technology) conjugated to horseradish peroxidase were incubated for 1 h at room temperature. The bands were visualized using the enhanced chemiluminescence (ECL) system and the gray densities were quantified with ImageJ software.

### Flow Cytometry

The apoptotic rate was measured by flow cytometry using an Annexin V-FITC/PI apoptosis assay kit (Beyotime, Shanghai, China). After PMNs culture for 6 h, the cells were resuspended in 500 mL binding buffer containing 5 mL Annexin V FITC and 10 mL PI, and incubated at room temperature in the dark for 20 min. The presence of apoptotic cells was analyzed by flow cytometry on a Beckman flow cytometer (CA, USA) within no more than 1 h.

### Statistical Analysis

The gray values of the protein electrophoresis bands were analyzed by ImageJ software (National Institutes of Health, Bethesda, MD, USA). The results are presented as the mean ± standard error of the mean and analyzed using SPSS 19.0 software (IBM Corp, Armonk, NY, USA). GraphPad Prism 8.0 was used for graphical analysis. Analysis of variance was performed to evaluate the differences among the groups while the *t*-test was used for between-group analysis. A *P*-value lower than 0.05 was considered statistically significant and a *P*-value lower than 0.01 was considered highly significant (^*^P < 0.05, ^**^P < 0.01).

## Results

### E2 Enhances PMNs Viability

**Figure 1:**
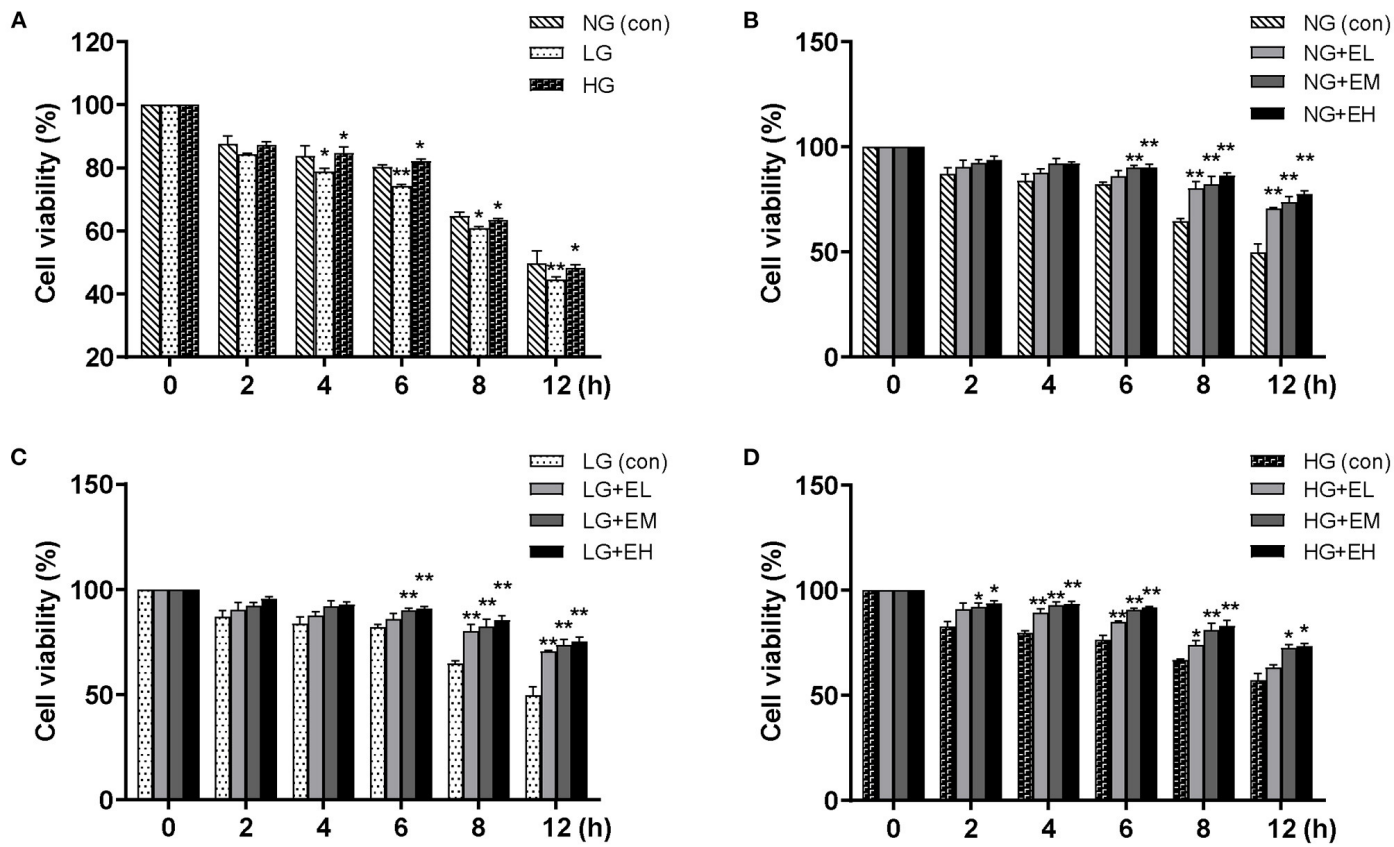
E2 enhances PMNs viability. PMNs were treated with NG (5.5 mM), LG (2.5 mM), HG (25 mM), NG+EL (20 pg/mL), NG+EM (200 pg/mL), NG+EH (450 pg/mL), LG+EL, LG+EM, LG+EH, HG+EL, HG+EM and HG+EH, for 0, 2, 4, 6, 8, and 12 h. **(A–D)** PMNs viability detected by CCK-8. The results are shown as the mean ± SD (*n* = 3). The *t*-test was used to analyze differences. ^*^P < 0.05, ^**^P < 0.01. The asterisk indicates a significant difference between the treatment group and the control group (con).

To investigate the effect of E2 on PMNs viability, the CCK-8 viability assay was used. The results showed that compared with NG, PMNs viability did not change significantly at 0–2 h in LG and HG; PMNs viability decreased significantly in LG group and increased significantly at 4–6 h in HG; PMNs viability decreased significantly at 8–12 h in both LG and HG (Figure 1A). E2, including low E2 (EL), moderate E2 (EM) and high E2 (EH) doses, significantly increased PMNs viability, especially enhancing viability after 6 h at low glucose (LG), normal glucose (NG) levels and high glucose (HG) levels (Figures 1B–D). These data indicated that E2 influences and maintains the viability of PMNs *in vitro*.

### E2 Promotes PMNs Glycogen Synthesis by Inhibiting GSK-3β Activity

**Figure 2:**
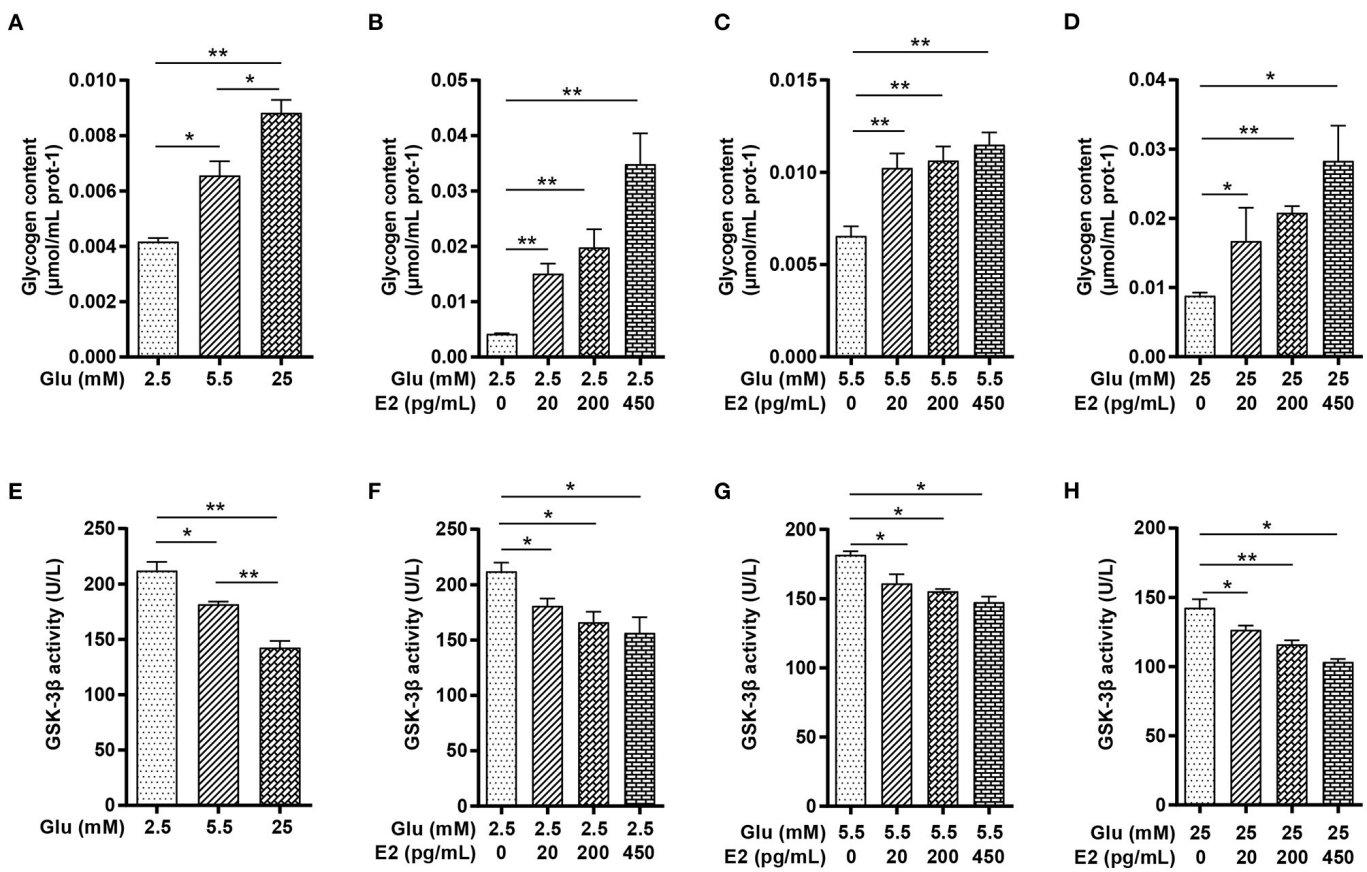
E2 promotes PMNs glycogen synthesis by inhibiting GSK-3β activity. PMNs was treated with LG, NG, HG, LG+EL, LG+EM, LG+EH, NG+EL, NG+EM, NG+EH, HG+EL, HG+EM and HG+EH for 6 h. **(A–D)** Glycogen content was determined by the biochemical method. **(E–H)** GSK-3β activity was determined by ELISA. The results are shown as the mean ± SD (*n* = 3). Differences were analyzed using the t-test. ^*^P < 0.05, ^**^P < 0.01. The significant difference between the two groups is indicated by a line and an asterisk.

To understand the glucose metabolism of PMNs in different glucose conditions and the possible regulatory role of different concentrations of E2, we first measured the glycogen content of the cells by biochemical methods and GSK-3β activity by ELISA. The results showed that the glycogen content was significantly increased, whereas the GSK-3β activity decreased in a concentration-dependent manner with the glucose levels (Figures 2A,E). E2 significantly increased the glycogen content, and this increase was related to the E2 concentration in LG, NG and HG in a dose-dependent manner (Figures 2B–D) while GSK-3β activity decreased significantly with increasing E2 concentration at LG, NG and HG (Figures 2F–H). These results suggested that EH strongly promotes glycogen synthesis through the inhibition of GSK-3β activity.

### E2 Regulates the Activity of PFK1 and G6PDH and Maintains ATP Homeostasis in PMNs

**Figure 3:**
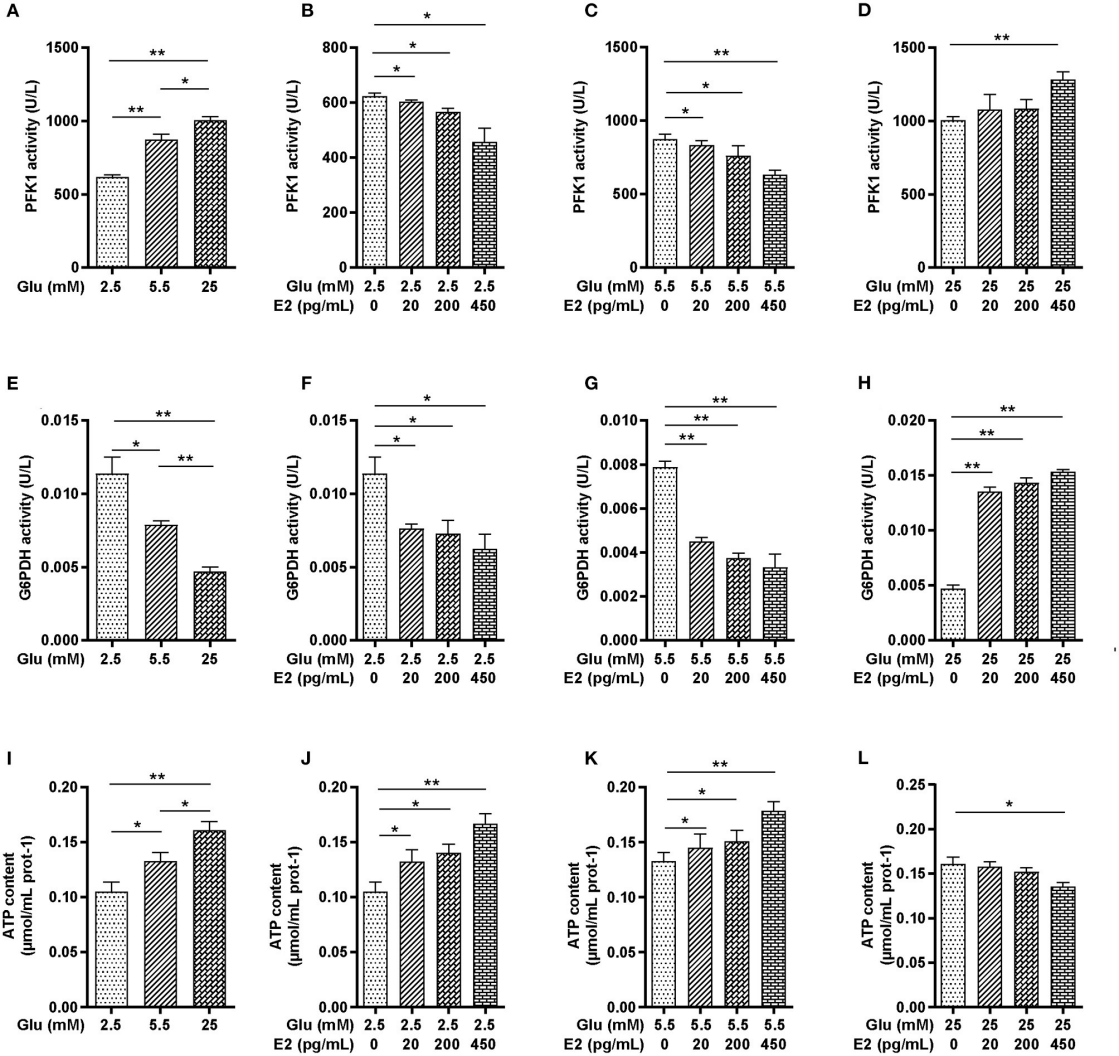
E2 regulates the activity of PFK1 and G6PDH and maintains ATP homeostasis in PMNs. PMNs were treated with LG, NG, HG, LG+EL, LG+EM, LG+EH, NG+EL, NG+EM, NG+EH, HG+EL, HG+EM and HG+EH for 6 h. **(A–D)** PFK1 activity was determined by ELISA. **(E–H)** G6PDH activity was determined by biochemical methods. **(I–L)** ATP contents were determined by biochemical methods. The results are shown as the mean ± SD (*n* = 3). The significance of the difference was analyzed by the t- test. ^*^P < 0.05, ^**^P < 0.01.

To determine the action of E2 on increased glycogen content in PMNs, we focused on glycolysis and the PPP in glucose metabolism pathways which provide ATP to meet the requirements of glycogen synthesis and energy expenditure in PMNs. The results showed that PFK1 activity was significantly increased in a glucose-dependent manner (Figure 3A). Under LG and NG conditions, EL, EM and EH significantly decreased PFK1 activity in a time-dependent manner, while under HG conditions, only EH significantly increased PFK1 activity (Figures 3B–D). The activity of G6PDH decreased in a dose-dependent manner with increasing glucose concentration (Figure 3E), specifically, decreasing with increasing E2 concentration at both NG and LG (Figures 3F,G) while showing the opposite effect at HG (Figure 3H). The results showed that EH was able to promote or inhibit PFK1 and G6PDH, the key catabolic enzymes of cellular glucose in PMNs under different glucose conditions. In addition, the results showed that the ATP content increased significantly in a glucose-dependent manner (Figure 3I), increasing with increased E2 concentrations at LG and NG (Figures 3J,K), while decreasing in response to E2 at HG and with the lowest level at EH (Figure 3L). These results indicated that EH had significant effects on cellular glucose catabolism by regulation of the activity of PFK1 and G6PDH, and on ATP production. Therefore, the EH concentration was used for the following experiments.

### E2 Regulates Glucose Uptake and Utilization by Regulating Glucose Transporters Expression and HK Activity

**Figure 4:**
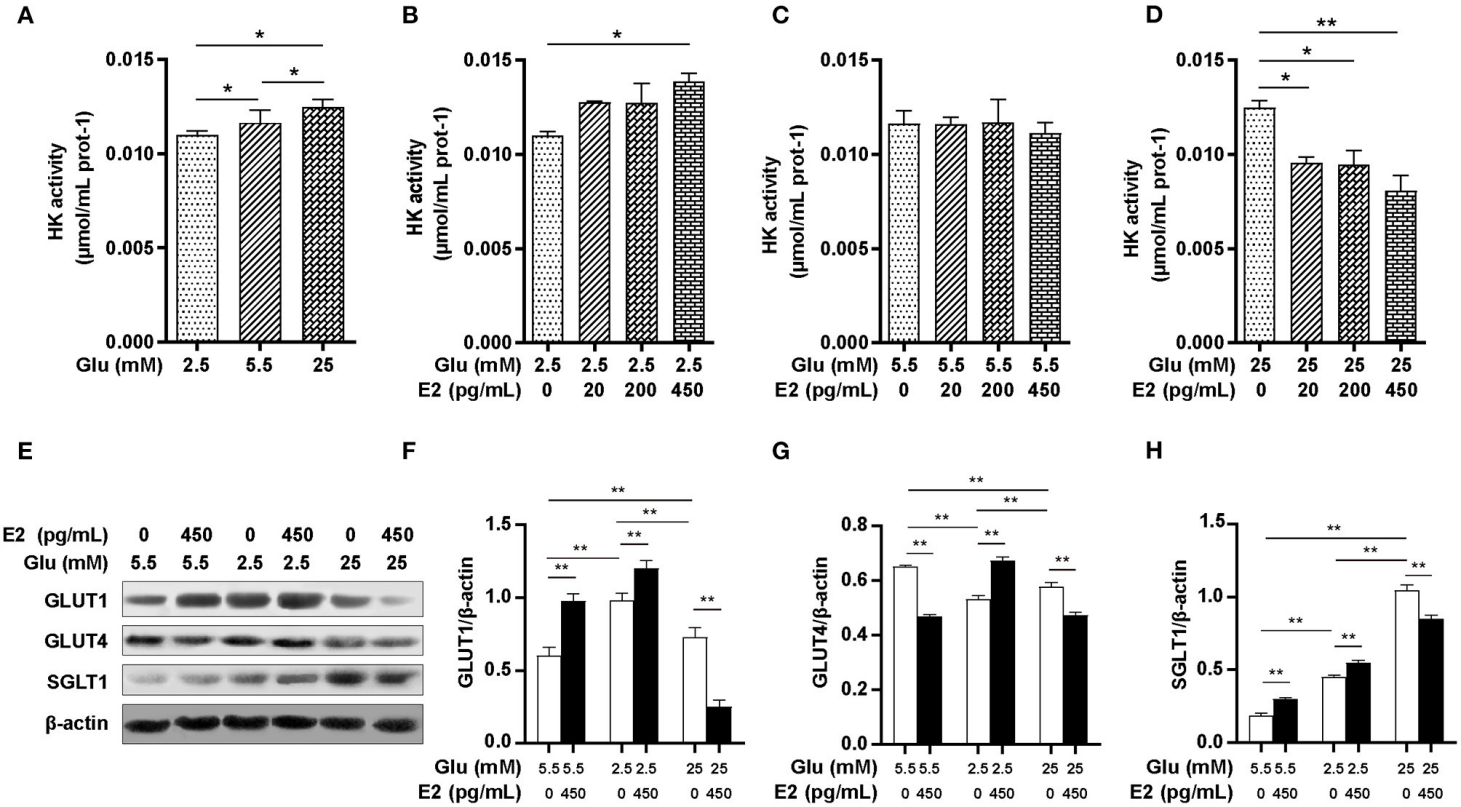
E2 regulates glucose uptake and utilization by regulating the activities of HK and glucose transporters. PMNs were treated with LG, NG, HG, LG+EL, LG+EM, LG+EH, NG+EL, NG+EM, NG+EH, HG+EL, HG+EM and HG+EH for 6 h. **(A–D)** HK activity was measured by the biochemical method. **(E–H)** The expression of GLUT1, GLUT4 and SGLT1 were detected by WB, and β-actin was used as an internal control. The results are shown as the mean ± SD (*n* = 3). The significance of the difference was analyzed using the *t*-test. ^*^P < 0.05, ^**^P < 0.01.

HK is a key enzyme of glucose catabolism: once glucose has been taken up by transporters from the extracellular environment, HK transforms the absorbed glucose into G6P to provide substrates for glycogen synthesis, glycolysis or the PPP. To identify the specific role of E2 on glucose uptake and utilization, we analyzed the expression of GLUT1, GLUT4 and SGLT1 by WB and HK activity by biochemical measurement. The results showed that HK activity increased in response to glucose in a dose-dependent manner (Figure 4A). EL and EM did not increase HK activity while EH significantly enhanced HK activity at LG (Figure 4B). Although E2 had no significant effect on HK activity at NG (Figure 4C), HK activity was decreased in an E2 concentration-dependent manner at HG (Figure 4D). The WB results showed that compared with NG, the expression of GLUT1 and SGLT1 were significantly increased and GLUT4 expression was significantly decreased at LG and HG levels (Figures 4E–H). E2 thus promoted the expression of GLUT1, GLUT4 and SGLT1 at LG while, in contrast, inhibiting expression at HG. At NG levels, E2 promoted the expression of both GLUT1 and SGLT1 while inhibiting GLUT4 expression. These results suggested that the regulation of glucose uptake and utilization by E2 depends on up-regulating or down-regulating the expression of transporters and HK activity, and that this is a crucial mechanism by which PMNs handle energy stress.

### E2 Regulates AMPK-Dependent Autophagy in PMNs

**Figure 5:**
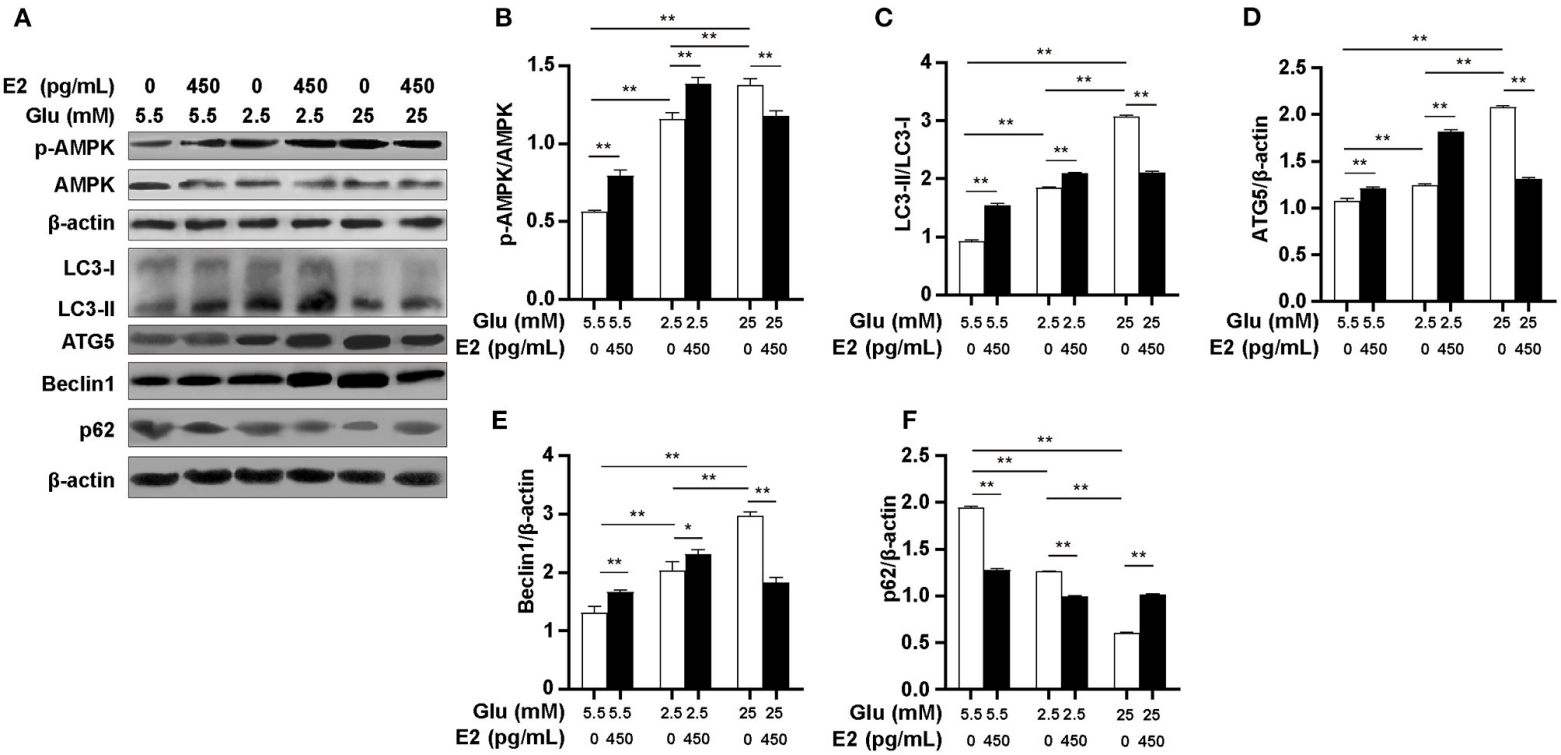
E2 regulates AMPK-dependent autophagy in PMNs. PMNs were treated with NG, NG+EH, LG, LG+EH, HG and HG+EH for 6 h. **(A–F)** Expressions of LC3, ATG5, Beclin1, p62, p-AMPK and AMPK were analyzed by WB with β-actin used as an internal control. The results are shown as the mean ± SD (*n* = 3). Differences were analyzed using the *t*-test.^*^P < 0.05, ^**^P < 0.01.

To determine whether the variation in ATP content under different glucose conditions is the result of pathways other than glycolysis and the PPP, we further investigated the effect of E2 on the autophagy pathway of AMPK and the expression of autophagy-related proteins LC3, ATG5, Beclin1 and p62 by WB. The results indicated that the p-AMPK/AMPK ratio at both LG and HG was significantly higher than at NG, and was significantly higher at HG than that at LG (Figures 5A,B). E2 significantly increased the level of AMPK phosphorylation at LG and NG but decreased it at HG. The expression of LC3, ATG5 and Beclin1 was increased while that of p62 was reduced at LG and HG rather than at NG, especially this expression was most significant at HG (Figures 5A,C–F). E2 significantly increased the expression of LC3, ATG5 and Beclin1, and significantly decreased p62 at LG and NG, but showed the opposite results at HG. These results suggested that E2 can maintain the optimal concentration of ATP by regulating autophagy according to glucose environment and intracellular ATP level, which may provide a material guarantee for maintaining cell survival and the development of immune potential in PMNs.

### E2 Inhibits PMNs Apoptosis

**Figure 6:**
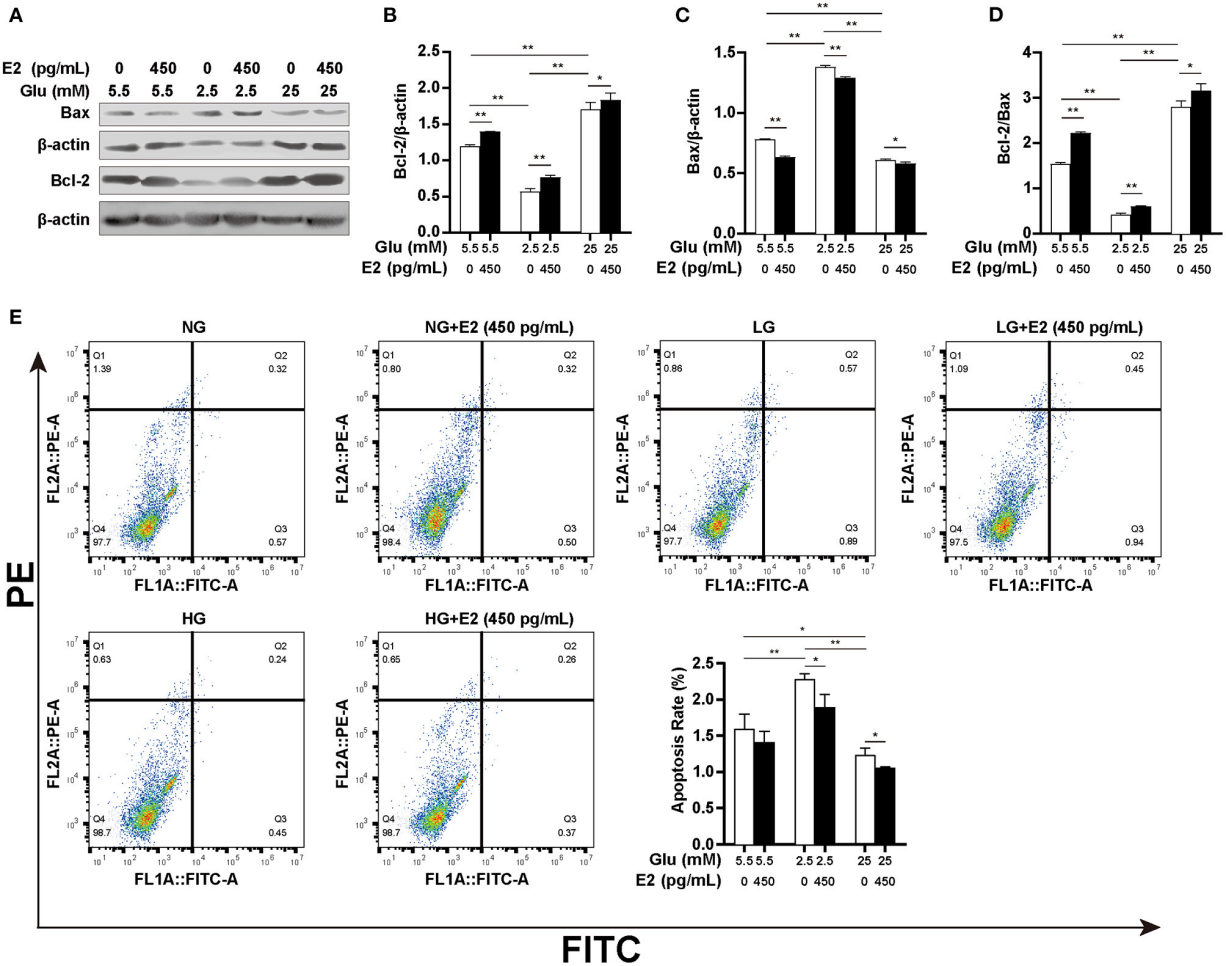
E2 inhibits PMNs apoptosis. PMNs were treated with NG, NG+EH, LG, LG+EH, HG and HG+EH for 6 h. **(A–D)** The expression of the apoptosis-related proteins Bax and Bcl-2 and the Bcl-2/Bax ratio were analyzed by WB. **(E)** Apoptosis rates in PMNs were assayed by flow cytometry. The results are shown as the mean ± SD (*n* = 3). Differences were analyzed using the *t*-test. ^*^P < 0.05, ^**^P < 0.01.

Low ATP levels in apoptotic PMNs indicate the importance of the relationship between spontaneous apoptosis and autophagy for cell survival. We, therefore, verified the effect of E2 on PMNs apoptosis under different glucose conditions by WB and flow cytometry. The results showed that at LG, there was a significantly higher Bax expression, lower Bcl-2 expression and Bcl-2/Bax ratio (Figures 6A–D) and an increased apoptosis rate compared to NG (Figure 6E), but there was an opposite effect at HG. However, E2 significantly increased the Bcl-2/Bax ratio and decreased the apoptosis rate at LG and HG. Our results suggested that E2 can protect PMNs by inhibiting apoptosis resulting from the environmental stress of lack or excess of glucose. In other words, the inhibition of apoptosis by E2 requires the change of autophagy level to maintain cell survival, and the occurrence of autophagy and apoptosis is based on the changes in glucose intake and metabolism.

## Discussion

We found that E2 promotes glycogen storage by promoting glycogen synthesis and maintains ATP homeostasis by enhancing glucose uptake and regulating autophagy in the context of changing extracellular glucose levels. These results suggested that E2 exerts a significant effect on both glucose uptake and utilization and, in particular, plays an important role in sustaining cell viability and promoting the glycogen storage and ATP content in situations of glucose deficiency in cattle PMNs *in vitro*. Therefore, E2 may be a key factor in maintaining the viability and enhancing the immune potential of PMNs for fulfilling immune function in periparturient cattle suffering from NEB.

Vazquez-Anon et al. (29) observed that hypoglycemia in periparturient cattle affects chemotaxis, phagocytosis, and killing capacity in PMNs due to reduced glycogen storage. Similarly, hyperglycemia during calving also impairs PMNs function and increases the risk of postpartum infection (30). Our results showed that PMNs viability was significantly affected by both low and high glucose, and the higher glucose condition significantly increased the PMNs glycogen content. Galvao et al. (31) found that treatment with glucose narrowed the difference in PMNs viability between cows suffering from uteritis and healthy cows, suggesting that hypoglycemia during the perinatal period is highly correlated with PMNs dysfunction and leads to disease susceptibility. It is well known that the E2 concentrations in perinatal cattle typically change from basal to peak and back to basal again. Our findings showed that different E2 concentrations significantly enhanced the viability of PMNs in low or high glucose environments *in vitro*, and we speculate that E2 exerts an active action on maintaining PMNs viability by regulating cellular glucose metabolism.

To further understand the E2 regulatory mechanism on PMNs glucose metabolism, we first evaluated the effect of E2 on PMNs glycogen synthesis. As the main energy source in PMNs, the glycogen content mostly depends on extracellular glucose uptake, and glucose deficiency leads to glycogen reduction in PMNs (31). Although previous studies have confirmed that E2 can promote utero glycogen synthesis in rats and rabbits (8, 32), the effect of E2 on glycogen synthesis in PMNs has been less studied. Our study showed that E2 promotes glycogen synthesis in cattle PMNs under different glucose concentrations *in vitro*, therefore, may benefit PMNs function in periparturient cattle suffering from NEB. ERα is the specific receptor for E2 and is a substrate of GSK-3β. E2 inhibits GSK-3β activity depending on the detachment of ERα from the ERα/GSK-3β complex (33). Reports have shown that E2 activates uterine epithelial cell proliferation by inhibiting GSK-3β-induced PI3K pathway activation (34). Our results showed that glycogen content depends on increased glucose concentration, and that E2 enhanced glycogen synthesis and inhibited GSK-3β activity in PMNs. Recent studies have shown that in mouse PMNs, the glycogen cycle can be used to produce energy at inflammatory sites where nutrients are limited (35). This suggested that moderate and well-timed amounts of E2 may contribute to the maintenance of the immune potential of PMNs in periparturient cattle by increasing the glycogen storage.

In terms of the glucose absorbed from the environment in PMNs, one part is stored in the form of glycogen while the other is competitively utilized between the PFK1-mediated glycolysis pathway and the G6PDH-mediated PPP. In mouse PMNs incubated with 25 mM glucose, there was a 50% dose-dependent reduction in G6PDH and oxygen production (36), and G6PDH deficiency in PMNs from diabetic mice resulted in reduced germicidal capacity and peroxide production (37). Therefore, we examined the activities of PFK1 and G6PDH in cattle PMNs and found that G6PDH activity decreased in response to increased glucose concentration. Addition of E2 to low- or normal-glucose medium attenuated G6PDH activity while E2 addition to high-glucose medium enhanced G6PDH activity, indicating that the impact of E2 on the PPP is involved in the extracellular glucose level. Newsholme et al. (38) found that the addition of glucose increased enzyme activity in glycolysis and ATP production in PMNs. Similarly, this study found that increased extracellular glucose enhanced PFK1 activity and ATP production. However, E2 significantly inhibited PFK1 activity and increased ATP content under low- or normal-glucose conditions, and enhanced PFK1 activity, and decreased ATP content under high-glucose conditions. These results raise an interesting question: E2 has been found to promote glycogen synthesis by inhibition of PFK1, G6PDH, and GSK-3β activity under low- or normal-glucose conditions, which ought to reduce the ATP content, however, our results showed an increase in ATP content. To answer this question, we next evaluated HK activity, glucose transporter expression, and autophagy levels in cattle PMNs.

Evidence has suggested that glucose uptake by cells is the result of functional coupling between GLUTs and HK (39). GLUT1 is expressed and up-regulated in a glucose-rich environment and mediates extracellular glucose uptake in mouse (40) and human (41) PMNs, and GLUT1, but not GLUT4, is regarded as the key link between numerous factors regulating glucose uptake in PMNs. However, we have not seen the report regarding the E2-mediated GLUT1 in PMNs, although E2 could up-regulate GLUT1 expression in breast cancer cells (42). Our study demonstrated that GLUT1, GLUT4, and SGLT1 protein are expressed in cattle PMNs. Low- and high-glucose promoted the expression of GLUT1 and SGLT1 and inhibited GLUT4 expression. We observed that the enhanced glucose uptake depends mainly on GLUT1 and SGLT1 in low-glucose environments, and which only relies on SGLT1 in the high-glucose environment. E2 enhanced the expression of GLUT1/4 and SGLT1 at low glucose levels, and inhibited GLUT4 expression at normal and high glucose levels, whereas E2 promoted SGLT1 expression at low- and normal-glucose levels and inhibited SGLT1 at high glucose levels. This demonstrates that E2 enhanced glucose uptake by promoting the expression of SGLT1 and GLUT1/4 in low glucose environments and preventing excessive glucose uptake by inhibiting SGLT1 and GLUT1/4 in high glucose environments. Thus, our results obviously defined that the promoting or inhibiting glucose uptake by E2 depends on glucose transporters function relating with the extracellular glucose level. Meanwhile, we found that HK activity was positively correlated with the level of environmental glucose and that HK activity was promoted by E2 under low glucose conditions while remaining unchanged under normal glucose conditions and being inhibited under high glucose conditions. This further provided the direct evidence for revealing glycogen increase in the perspective of intracellular glucose metabolism. Clearly, E2 enhanced the PMNs glycogen storage by upregulating glucose uptake, which lies in HK activity and the level of GLUT1/4 and SGLT1 expression in different glucose environments. Considering the process of glycogen synthesis, by its nature, needs to consume a large amount of ATP, here raises an interesting question of where the increased ATP come from when the activity of PFK1, G6PDH, and GSK-3β are inhibited by E2 in low- or normal-glucose environments in this study. Previous studies showed that activated AMPK enhance the plasma membrane localization of GLUT1 and GLUT4 in skeletal muscle (43) and leads to SGLT1 upregulation and glucose uptake promotion in cardiomyocytes (44). These finding remind us to evaluate AMPK activation may help to uncover the underlying relevance of E2 to cellular ATP content.

We observed that activated AMPK is synchronized with the increased glucose uptake, E2 promoted AMPK phosphorylation in the low- and normal-glucose environment, but inhibited AMPK activation under high-glucose conditions. This indicates that E2 can regulate ATP levels. Some studies have found that inhibiting glycolysis-dependent ATP production could activate AMPK (45) and activation of AMPK could increase the ATP level in ovarian cancer cells (46). Correspondingly, our results showed that E2 increased the ATP content by inhibiting PFK1 and activating AMPK at low and normal glucose levels, and yet E2 reduced the ATP content by enhancing PFK1 activity and inhibiting AMPK at high glucose levels. Therefore, it is reasonable to speculate that the change in ATP level resulting from addition of E2 at different glucose levels may depend on AMPK activity, and the increased ATP may be derived from an AMPK-dependent pathway, such as the autophagy pathway. We, therefore, next investigated autophagy-related proteins to answer this question.

Activation of autophagy at low glucose levels helps to maintain cellular energy homeostasis, whereas several identified signaling pathways also activate autophagy at high glucose levels (47). Ma et al. found that high glucose (20 mM) induced podocyte autophagy and damage by enhancing the expression of LC3 and Beclin1 proteins (48). Similarly, our study showed that autophagy was promoted in PMNs under low- and high-glucose conditions. The evidence showed that E2 could protect the vascular endothelium by promoting autophagy (49), but E2 protects cardiomyocytes from LPS damage by inhibiting autophagy (50). The controversial results may lies in the different conditions or cells. Our results suggested that E2 enhanced autophagy in low- or normal-glucose conditions and inhibited autophagy under high glucose conditions by regulating AMPK activity in PMNs *in vitro*. It may indicate that E2 promotes autophagy during energy deficiency or prevent damage to the cells by inhibiting autophagy in nutrient-rich environments. Therefore, the glucose environment is likely to be an essential prerequisite for E2-regulated autophagy for cattle PMNs. As known that glycolysis is the main pathway of ATP production in PMNs, whereas in the case of limited glucose supply, PMNs also can obtain the required energy through fatty acid oxidation (36). In human PMNs, autophagy provides sufficient free fatty acids through the decomposition of lipid droplets and supports the FAO-OXPHOS pathway to produce more ATP (51). This verifies our results from another aspect, that is, E2 may promote lipophagy to maintain a steady-state ATP content in cattle PMNs under deficient glucose conditions.

Both apoptosis and autophagy are necessary for PMNs survival, and the ATP content is lower in apoptotic PMNs (52), while E2 can protect mouse pancreatic β cells from apoptosis through the ERα mechanism (53). To further understand how E2 protects PMNs from spontaneous apoptosis, we analyzed the expression of autophagy- and apoptosis-related proteins Bcl-2 and Bax and the apoptosis rate. The results showed that low glucose promoted apoptosis and high glucose inhibited apoptosis. This indicated that autophagy is not sufficient to counteract spontaneous apoptosis under low-glucose conditions. The lower apoptosis rate in high-glucose, not in low-glucose conditions, could be attributed to enough ATP production resulting from increased autophagy and glycolysis being sufficient to inhibit spontaneous apoptosis. Moreover, our study also found that E2 inhibited apoptosis accompanied by inhibition of GSK-3β activity in PMNs under different glucose conditions, suggesting that E2-induced GSK-3β inactivation is the initial point of regulating glucose metabolism and the root cause of triggering or inhibiting lipophagy.

## Conclusion

In conclusion, we demonstrated that E2 promotes glycogen synthesis and increases the ATP content of cattle PMNs by enhancing the activity of HK, expression of GLUT1/4 and SGLT1, and the level of autophagy, as well as by inhibiting the activity of PFK1, G6PDH and GSK-3β under conditions of glucose restriction. This finding suggested that the molecular mechanisms by which E2 controls cellular energy levels is essential for protecting the cells from apoptosis and reinforcing the immune potential of PMNs. More research is required to further elucidate the mechanism by which E2 regulates glucose metabolism. This study provided a meaningful understanding of the effects of E2 on PMNs function in periparturient cattle suffering from NEB.

## Data Availability Statement

The original contributions presented in the study are included in the article/Supplementary Materials, further inquiries can be directed to the corresponding authors.

## Ethics Statement

The animal study was reviewed and approved by Inner Mongolia Nationalities University Animal Care and Use Committee (no. SCXK-2020-0002).

## Author Contributions

XW and YZ performed the experiments and wrote the manuscript. YL and MT collected and analyzed the data. QD and JM revised the manuscript. LD revised the manuscript and supervised the entire project. All authors read and approved the final manuscript.

## Funding

This work was supported by National Natural Science Foundation of China (31760752 and 31260626). Natural Science Foundation of Inner Mongolia Autonomous Region (2018LH03009 and 2019LH03020). Young scientific and technological talents in Inner Mongolia (Inner Mongolia, China, Grant No. NJYT-20-B30).

## Conflict of Interest

The authors declare that the research was conducted in the absence of any commercial or financial relationships that could be construed as a potential conflict of interest.

## Publisher's Note

All claims expressed in this article are solely those of the authors and do not necessarily represent those of their affiliated organizations, or those of the publisher, the editors and the reviewers. Any product that may be evaluated in this article, or claim that may be made by its manufacturer, is not guaranteed or endorsed by the publisher.

## Glossary

NEB, Negative energy balance; PMNs, Polymorphonuclear neutrophils; E2, 17β-estradiol, estrogen; GLU, Glucose; NG, Normal concentration of glucose; LG, Low concentration of glucose; HG, High concentration of glucose; EL, Low concentration of estrogen; EM, Medium concentration of estrogen; EH, High concentration of estrogen; CCK-8, Cell counting kit-8; HK, Hexokinase; G6P, glucose 6-phosphate; PFK1, Phosphofructokinase-1; PPP, Pentose phosphate pathway; G6PDH, Glucose-6-phosphate dehydrogenase; GSK-3β, Glycogen synthase kinase-3β; ATP, Adenosine triphosphate; SGLT, Sodium-glucose co-transporter; GLUTs, Glucose transporters system; AMPK, AMP-activated protein kinase; ATG5, Autophagy-related gene 5; LC3, light chain 3; p62, Sequestosome-1; Bcl-2, B-cell lymphoma-2; Bax, Bcl-2-associated x; WB, Western blotting.
