# Supplementary figures and images for: Estrogen Regulates Glucose Metabolism in Cattle Neutrophils Through Autophagy
Source: Front Vet Sci. 2021 Nov 29;8:773514. doi: 10.3389/fvets.2021.773514 (PMC8666889; doi:10.3389/fvets.2021.773514)

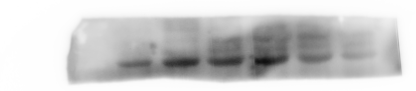

Supplement: Supplementary file 1 [file Data_Sheet_1.ZIP › original source data/Western blot image Figure 4E/1. First line GLUT1.tif]

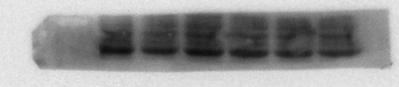

Supplement: Supplementary file 1 [file Data_Sheet_1.ZIP › original source data/Western blot image Figure 4E/2. Second line GLUT4.tif]

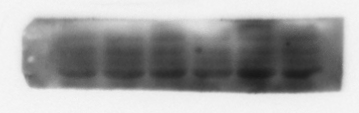

Supplement: Supplementary file 1 [file Data_Sheet_1.ZIP › original source data/Western blot image Figure 4E/3. Third line SGLT1.tif]

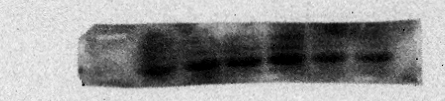

Supplement: Supplementary file 1 [file Data_Sheet_1.ZIP › original source data/Western blot image Figure 4E/4. Fourth line a┬-actin.tif]

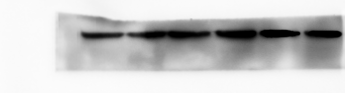

Supplement: Supplementary file 1 [file Data_Sheet_1.ZIP › original source data/Western blot image Figure 5A/1 line p-AMPK.tif]

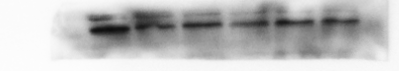

Supplement: Supplementary file 1 [file Data_Sheet_1.ZIP › original source data/Western blot image Figure 5A/2 line AMPK.tif]

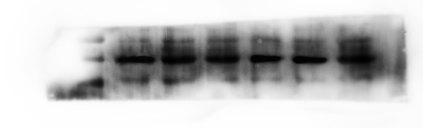

Supplement: Supplementary file 1 [file Data_Sheet_1.ZIP › original source data/Western blot image Figure 5A/3 line a┬-actin.tif]

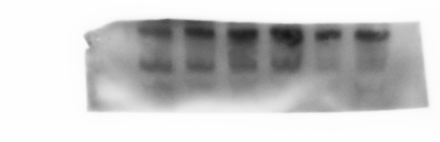

Supplement: Supplementary file 1 [file Data_Sheet_1.ZIP › original source data/Western blot image Figure 5A/4th line LC3.tif]

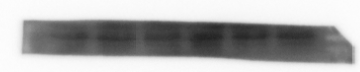

Supplement: Supplementary file 1 [file Data_Sheet_1.ZIP › original source data/Western blot image Figure 5A/5th line ATG5.tif]

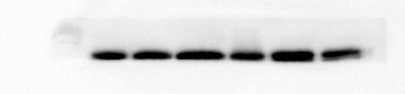

Supplement: Supplementary file 1 [file Data_Sheet_1.ZIP › original source data/Western blot image Figure 5A/6th line Beclin1.tif]

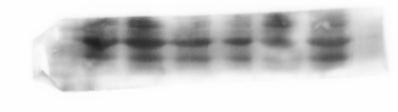

Supplement: Supplementary file 1 [file Data_Sheet_1.ZIP › original source data/Western blot image Figure 5A/7th line p62.tif]

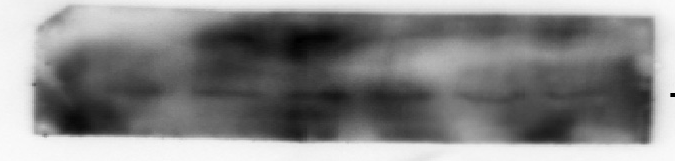

Supplement: Supplementary file 1 [file Data_Sheet_1.ZIP › original source data/Western blot image Figure 6A/1. First line Bax.tif]

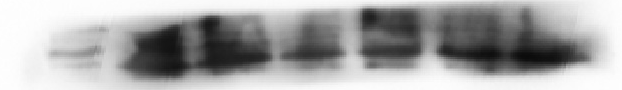

Supplement: Supplementary file 1 [file Data_Sheet_1.ZIP › original source data/Western blot image Figure 6A/2. Second line a┬-actin.tif]

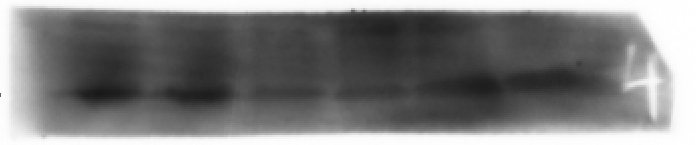

Supplement: Supplementary file 1 [file Data_Sheet_1.ZIP › original source data/Western blot image Figure 6A/3. Third line Bcl-2.tif]

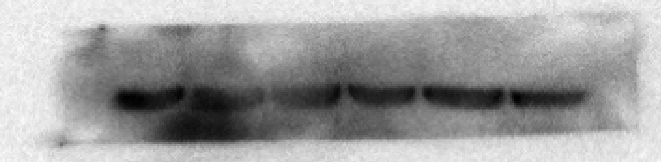

Supplement: Supplementary file 1 [file Data_Sheet_1.ZIP › original source data/Western blot image Figure 6A/4. Fourth line a┬-actin.tif]
